# Peer Review of “Patterns of Physical Activity Among University Students and Their Perceptions About the Curricular Content Concerned With Health: Cross-sectional Study”

**DOI:** 10.2196/37119

**Published:** 2022-04-29

**Authors:** Viktoria Nagy

**Affiliations:** 1 Faculty of Informatics Eötvös Loránd University Budapest Hungary

**Keywords:** physical activity, university students, university, exercise, students, inactive, curricula, healthy lifestyle, higher education

*This is a peer-review report submitted for the paper “Patterns of Physical Activity Among University Students and Their Perceptions About the Curricular Content Concerned With Health: Cross-sectional Study.”*

## Round 1 Review

### General Comments

This paper [1] was about the physical activity pattern of university students aiming at measuring this for the first time systematically as well as creating a new tool in order to have more accurate results. The authors collected a large body of data over several years, which gives an accurate and realistic perspective of the physical activity patterns of university students in India. It was an honour to read this remarkable job the authors did over the years.

### Specific Comments

1. I find the Introduction part quite short compared to the literature mentioned in the Discussion. I learned more about the literature from the Discussion than from the Introduction. I’d suggest writing a slightly longer introduction with details on activity patterns of different age groups. This could also point to the missing age group data this paper focuses on.

2. The authors mention in the first paragraph of the introduction “an increased engagement with video games, cell phones, television, computers, and social media are possibly some of the important contributing factors to this trend among youth.” I’d write in more detail about this or have a bigger emphasis on this perspective in the paper, both in the introduction and in the discussion, as the manuscript was submitted to the Journal of Medical Internet Research.

3. The authors mention in Methods, in the study design and sampling, “time and other limitations.” I’d rather mention these in the limitations part of the Discussion, and I’d explicitly say what the other limitations not listed here are. The authors write “approximately 4600 students” in this section. On the other hand, I read the exact number later on. I’d suggest writing the exact number because it is accessible.

4. In the “translation and revalidation” subheading, the authors mention “professional” who did the translation and retranslation. I find it important to expand what kind of professionals they were? Translators, interpreters, psychologists, English teachers, or what profession did they have? You also mention “suitable corrections were made.” What does this mean? Were certain items deleted based on a set of criteria? I am not sure I understand the last sentence “both the versions of the tool were used in the study to collect data based on student preference.” I wonder if it would be possible to make it clear what two versions were used?

5. In the “development of a new tool,” I was wondering in what language did you state these questions? My understanding is that in Hindi. I’d suggest writing it explicitly if so. I also wonder why these 5 items were used? what was the process of creating these items? Were there possibly more and then you deleted the ones that did not work? What did you base your decision on to use these exact 5 items?

6. In the “validation of the new tool” you write “acceptable range.” I suggest giving a literature reference on what you based your decision on, what is acceptable, and what is not. I read the manuscript and you reported the Cronbach alpha. In my understanding, this means the tool is reliable; however, it was not validated. For example, correlation with other tools.

7. The authors reported the data collection was between 2016 and 2019. This is a long stretch of time, and physical activity patterns can change in different groups year by year. I’d suggest for the authors to consider a statistical analysis on the data year by year. For example, people who filled out the questionnaire in 2016, the ones in 2017, and so on.

8. I read in the results you reported significant and not significant results. I’d consider writing a sentence about the direction of significant results. For example, “the difference between physical activity of students of different age groups was statistically significant.” I’d find it useful to read a sentence about which age group was more active and which one less active.

9. I’d find it useful if I could read the results in hour as well, besides reading them in minutes. As far as I understand, the tool used reports in minutes. However, it would be easier to read if I could read it also in hours.

10.I’d suggest using the last sentence of the results in the Discussion. “Hence, it can be presumed that the students in these faculties receive some or other kind of motivation to lead a physically active lifestyle as a part of their curriculum.

11. The authors write in the Discussion, “this is possibly one of the first studies from India that looks at psychical activity…”. I’d suggest not to use the phrase “possibly.” After having read the literature in India about psychical activity of students, it can be said if this is the first or one of the first papers reporting on the matter.

12. I’d find it useful to have a section for abbreviations.
